# Transcriptomics and methylomics of CD4-positive T cells in arsenic-exposed women

**DOI:** 10.1007/s00204-016-1879-4

**Published:** 2016-11-12

**Authors:** Karin Engström, Tomasz K. Wojdacz, Francesco Marabita, Philip Ewels, Max Käller, Francesco Vezzi, Nicola Prezza, Joel Gruselius, Marie Vahter, Karin Broberg

**Affiliations:** 1 Department of Laboratory Medicine, Division of Occupational and Environmental Medicine, Lund University, 221 85 Lund, Sweden; 2 Unit of Metals and Health, Institute of Environmental Medicine, Karolinska Institutet, Box 210, 171 77 Stockholm, Sweden; 3 Unit of Computational Medicine, Center for Molecular Medicine, Karolinska University Hospital, L805, 171 76 Stockholm, Sweden; 4 Bioinformatics Infrastructure for Life Sciences, Karolinska Institutet, 171 65 Solna, Sweden; 5 Science for Life Laboratory (SciLifeLab), Department of Biochemistry and Biophysics, Stockholm University, 106 91 Stockholm, Sweden; 6 Science for Life Laboratory, School of Biotechnology, Division of Gene Technology, Royal Institute of Technology, 171 65 Solna, Sweden; 7 Department of Mathematical and Computer Science, University of Udine, 331 00 Udine, Italy; 8 Aarhus Institute of Advanced Studies, Aarhus University, 8000 Aarhus C, Denmark

**Keywords:** Arsenic, Transcriptomics, Methylomics, CD4 cells, Immune system, Immunotoxic

## Abstract

Arsenic, a carcinogen with immunotoxic effects, is a common contaminant of drinking water and certain food worldwide. We hypothesized that chronic arsenic exposure alters gene expression, potentially by altering DNA methylation of genes encoding central components of the immune system. We therefore analyzed the transcriptomes (by RNA sequencing) and methylomes (by target-enrichment next-generation sequencing) of primary CD4-positive T cells from matched groups of four women each in the Argentinean Andes, with fivefold differences in urinary arsenic concentrations (median concentrations of urinary arsenic in the lower- and high-arsenic groups: 65 and 276 μg/l, respectively). Arsenic exposure was associated with genome-wide alterations of gene expression; principal component analysis indicated that the exposure explained 53% of the variance in gene expression among the top variable genes and 19% of 28,351 genes were differentially expressed (false discovery rate <0.05) between the exposure groups. Key genes regulating the immune system, such as tumor necrosis factor alpha and interferon gamma, as well as genes related to the NF-kappa-beta complex, were significantly downregulated in the high-arsenic group. Arsenic exposure was associated with genome-wide DNA methylation; the high-arsenic group had 3% points higher genome-wide full methylation (>80% methylation) than the lower-arsenic group. Differentially methylated regions that were hyper-methylated in the high-arsenic group showed enrichment for immune-related gene ontologies that constitute the basic functions of CD4-positive T cells, such as isotype switching and lymphocyte activation and differentiation. In conclusion, chronic arsenic exposure from drinking water was related to changes in the transcriptome and methylome of CD4-positive T cells, both genome wide and in specific genes, supporting the hypothesis that arsenic causes immunotoxicity by interfering with gene expression and regulation.

**Electronic supplementary material:**

The online version of this article (doi:10.1007/s00204-016-1879-4) contains supplementary material, which is available to authorized users.

## Introduction

Inorganic arsenic occurs in drinking water worldwide and in certain foods, mainly rice-based products. Long-term exposure to inorganic arsenic is a strong risk factor for different types of cancers as well as numerous non-cancer conditions, such as cardiovascular and lung diseases (IARC 2012). More recently, studies have identified immunotoxic effects of arsenic. For example, studies on newborns and children found that exposure to arsenic during pregnancy was associated with immunosuppressive effects, such as decreased thymus size and impaired thymic function, altered cytokine expression, and increased susceptibility to infections (Moore et al. 2009; Raqib et al. 2009; Ahmed et al. 2011, 2012, 2014; Rahman et al. 2011; Farzan et al. 2016). Arsenic-exposed adults and children had reduced proliferation of lymphocytes, fewer CD4-positive T cells, altered CD4 + :CD8 + T cell ratio, and fewer T-regulatory cells (Biswas et al. 2008; Hernandez-Castro et al. 2009; Kile et al. 2014; Nadeau et al. 2014). A few studies have reported that arsenic exposure may also increase the risk of allergies and autoimmune diseases (Tseng 2004; Soto-Pena et al. 2006). These findings suggest that arsenic exposure could result in both immune suppression and immune stimulation.

The mechanisms behind the immunotoxic effects of arsenic remain unclear (Ahmed et al. 2014; Ferrario et al. 2016). One potential mechanism is that arsenic could interfere with the expression of immune-related genes. This could result in failure to maintain homeostasis in the immune response to foreign antigens, potentially leading to immune dysfunction and immune-related diseases. In support of such a mechanism, microarray-based epidemiological studies have shown that arsenic exposure is associated with altered expression of immune-related genes in whole blood (Argos et al. 2006; Andrew et al. 2008; Salgado-Bustamante et al. 2010). The mode of action by which arsenic changes gene expression could, as recently indicated, occur by disturbing epigenetic processes that regulate gene expression, such as DNA methylation (Pilsner et al. 2012; Koestler et al. 2013; Broberg et al. 2014; Kile et al. 2014; Cardenas et al. 2015; Rojas et al. 2015).

We hypothesized that chronic arsenic exposure affects gene expression via alteration of DNA methylation of central components of the immune system. To evaluate this, we performed next-generation sequencing (NGS) of the transcriptome (by RNA sequencing) and the methylome (by target-enrichment NGS) of primary CD4-positive T cells, key immune cells in the defense against infections, in individuals chronically exposed to inorganic arsenic via drinking water.

## Methods

### Study participants, CD4-positive cell enrichment, and measurement of arsenic

This study involved women living in San Antonio de los Cobres and surrounding villages at around 4000 m above sea level in the Andes Mountains, Salta Province, Argentina. This area has varying concentrations of arsenic in the drinking water, but little industrial or traffic pollution (Concha et al. 2010). This population has been found to have an efficient metabolism of arsenic, i.e., they efficiently methylate inorganic arsenic to dimethylarsinic acid (DMA) with little excretion of the monomethylated metabolite, MMA, in urine (Vahter et al. 1995).

The study had 34 women enrolled in 2011. A detailed description of the study group has been published previously (Harari et al. 2013). Blood samples from the first 20 women recruited were sorted for CD4-positive T cells immediately after blood collection (K_2_EDTA tubes (Vacuette, Greiner Bio-One GmbH, Greiner, Germany), using the Dynabeads CD4 kit (Life Technologies, USA) and following the manufacturer’s protocol. Subsequently, RNA and DNA were extracted with the AllPrep DNA/RNA/Protein Mini kit (Qiagen, Hilden, Germany). Exposure to arsenic was assessed based on the sum concentration of metabolites of inorganic arsenic in urine (inorganic arsenic, MMA, and DMA), as measured by high-performance liquid chromatography (Agilent 1100 Series System, Agilent Technologies, Waldbronn, Germany) coupled with hydride generation and inductively coupled plasma mass spectrometry (Agilent 7500ce; Agilent Technologies, Tokyo, Japan) (HPLC-HG-ICPMS), as described previously (Concha et al. 2010). To correct for variation in urine dilution, we adjusted the obtained concentrations for the average specific gravity mean specific gravity of urine (1.020 g/mL), determined by a digital refractometer (EUROMEX RD 712 clinical refractometer; EUROMEX, Arnhem, the Netherlands). To obtain a large contrast in exposure, we selected samples from the eight women who showed the greatest differences in arsenic exposure (four with high exposure and four with lower exposure) and that had similar age and BMI. However, there was an overlap between the samples used for DNA and RNA analyses for only three of the women, due to limitations in sample amount. For the transcriptome analysis all samples were analyzed individually, and for the methylome analysis the samples in each exposure group were pooled. None of the women reported any disease or other health problems, and none reported drinking alcohol or smoking. The high- and low-exposure groups showed a fourfold to fivefold difference in urinary arsenic concentrations (Table S1).

### Transcriptome characterization: RNA sequencing (RNA-seq)

RNA concentration and purity were evaluated on a NanoDrop spectrophotometer, and RNA integrity (RIN) was evaluated on a Bioanalyzer 2100; the results showed good RNA quality (RIN > 7.5). Total RNA was used to prepare libraries, which were sequenced as paired-end, 100-bp reads on an Illumina HIseq 2500 (HiSeq Control Software 2.2.38/RTA 1.18.61). Library construction and sequencing were performed according to the manufacturer’s protocols by researchers at The Swedish National Center for Molecular Biosciences, Science for Life Laboratory (SciLifeLab), Stockholm, Sweden.

### Methylome characterization: target-enrichment NGS

Equal quantities of DNA from all four women (250 ng) for each exposure group were pooled and sequenced in two technical replicates. Sample processing included DNA quality control (using Qubit assays, from Life Technologies, USA, and Bioanalyzer, from Agilent, USA), target enrichment, bisulfite modification, library preparation, and sequencing. Target enrichment was performed with SureSelectXT Human Methyl-Seq kit (Agilent, USA), including the following genomic sequences likely to undergo changes in methylation: CpG islands, shores, and shelves, DNase I hypersensitive sites, Refseq genes, and Ensembl regulatory features. The enriched products were bisulfite treated with the Zymo EZ DNA Methylation Lightning kit (Zymo Research, USA) and sequenced by HiSeq 2500, in High Output mode 2 × 100 bp with the TruSeq SBS and the TruSeq PE Cluster kits, both v3 (Illumina, USA). The sample preparation was performed according to the manufacturers’ protocols by SciLifeLab.

### MS-HRM analysis

Methylation-sensitive high-resolution melting (MS-HRM) assays were designed and optimized as described previously (Wojdacz and Dobrovic 2007). LightCycler 480 (Roche, Germany) and LightCycler 480 High Resolution Melting Master (Roche) PCR chemistry were used.

### RNA-seq data analysis

RNA-seq reads were processed with TrimGalore (v0.3.7) for adapter removal and adaptive trimming. Alignment was performed with TopHat (v2.0.12), and reads were counted with HT-seq (v0.6.1) with GENCODE V19 GTF annotation. The features with zero counts were removed, and the size factor was estimated to normalize the libraries. The cumulative distribution of the counts before and after normalization was examined to check for proper normalization. Regularized log2 transformation (due to skewed data) was applied for further visualization and data exploration. Pairwise correlation and distance between samples were calculated and visualized, and principal component analysis (PCA) was used to explore sample clustering. The variability of gene expression was measured by coefficient of variation (standard deviation divided by the mean), and the variance explained was calculated on the top 20% variable genes ranked using the coefficient of variation. Differential expression analysis was performed using DESeq 2, contrasting high- vs. low-arsenic exposure groups. Differentially expressed genes (DEGs) were defined as having a log-twofold change in expression (log_2_FC) between arsenic exposure groups and with a false discovery rate (FDR) adjusted *p* value (q) <0.05. DEGs with a positive log-twofold change in the group with higher exposure, compared to the group with lower exposure, were defined as upregulated, while DEGs with a negative log-twofold change in the group with higher exposure compared to the group with lower exposure were defined as downregulated. Independent filtering was employed to calculate cutoff (<2.7) for the number of genes with low expression. There were 28,351 input genes, of which 11,326 (40%) showed low expression (<2.7) and 69 were defined as outliers (genes whose observed counts might not fit to a negative binominal distribution). Heatmaps were obtained for the DEGs. Enrichment for gene ontology was analyzed using TopGo with a Fisher test and the algorithm weight01, to take into account the structure of the gene ontology tree and to eliminate redundancy.

### Target-enrichment NGS data analysis

Adapter removal, adaptive trimming (quality score <28), and 5′ clipping (4 nucleotides) were performed using Trim Galore (v0.3.3). Trimmed sequences were mapped to the human genome (build hg19), de-duplication was performed, and methylation calls were extracted using Bismark (v0.10.0, with Bowtie2 v2.0.6) (Krueger and Andrews 2011). Downstream analysis was performed using bsseq (Hansen et al. 2012). CpGs with ≥10 × coverage in all samples were retained, and 2,705,455 CpGs were included in subsequent analysis. Genomic clusters of CpGs were identified: Regions covered by the capture probes were extended 100 bp on either side, and regions separated by <300 bp were merged into single clusters. To identify differentially methylated regions (DMRs), we first calculated a difference in methylation (∆Meth) for each CpG position between high- and low-exposure groups. The function “regionFinder” was used in the bumphunter package version 1.2.0 [modified from a previously published method (Jaffe et al. 2012)], providing the locations of the clusters and using a cutoff of ∆Meth = 10%. The DMRs were then filtered for those with at least four CpGs. DMRs with higher methylation in the high-exposure group compared to the lower-exposure group were defined as hypermethylated; DMRs with lower methylation in the high-exposure group compared to the lower-exposure group were defined as hypomethylated. For evaluation of technical reproducibility, SeqMonk (Zhao et al. 2014) was used to generate cumulative distribution plots that describe the methylation level at each CpG site versus a number of CpG sites with a given methylation level. The GREAT platform was used for analysis of gene ontology (McLean et al. 2010).

### Alignment RNA-seq and target-enrichment NGS

Gene overlap for DMRs and DEGs that were both statistically significantly associated with arsenic exposure group was further evaluated. DMRs included in these analyses were restricted to those in promoter regions, defined as within 500-base-pair downstream and 1500-base-pair upstream of the transcription start site.

## Results

### Descriptive data

We compared the transcriptomes and methylomes of CD4-positive T cells from four women with high-arsenic exposure (~300 µg/L in urine) to those from four women with lower-arsenic exposure (~60 µg/L; Table S1). The two exposure groups and the women evaluated for transcriptomes and methylomes showed no statistically significant differences in age, body mass index (BMI), or coca chewing (Table S1).

### Transcriptomics of CD4-positive T cells

We did not observe any bias in the distribution of number of transcripts, i.e., count data, with respect to exposure group, or any sample outliers when performing pairwise scatterplots of the samples (data not shown).

**Fig. 1:**
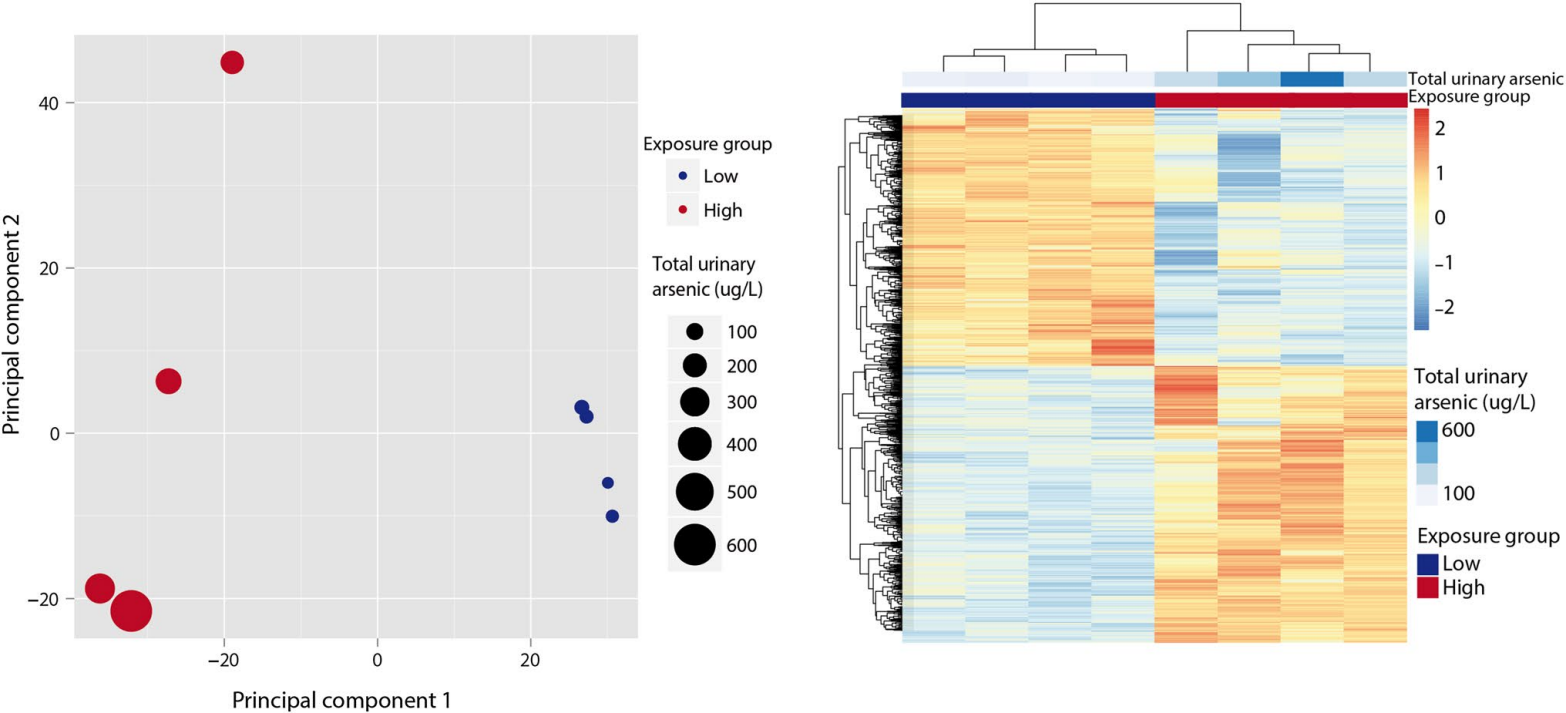
Principal components analysis (*left panel*) and heatmap (*right panel*) for differently expressed genes between high- and lower-arsenic exposure groups. In the principal components analysis, the first component separates samples based on their exposure group and explains 53% of the variance in gene expression for the top 20% variable genes. In the heatmap, the row Z-score of differentially expressed genes is plotted, which shows the relative change for each row (gene). Samples are correctly clustered according to the exposure group

We performed PCA to evaluate the genome-wide effects of arsenic on gene expression in CD4-positive T cells. The first principal component clearly separated samples based on their exposure group and explained 53% of the variance in gene expression among the top 20% variable genes (based on coefficient of variation) (Fig. 1, left panel). We identified multiple DEGs between arsenic exposure groups (Fig. 1, right panel): out of 28,351 input genes, the total fraction of DEGs, up- (9.7%, 2744 genes) or downregulated (9%, 2553 genes) in the high-arsenic group was 18.7%. We found the largest log_2_FC for downregulated genes: 79 genes showed a log_2_FC below −3, with a maximum of −4.7. For upregulated DEGs, 20 genes showed a log_2_FC above 3 with a maximum of 4.0.

Among the top 10 DEGs (based on *p* value), four have been linked to immune system function, and all were downregulated in the high-arsenic exposure group (Table S2): these genes encode tumor necrosis factor, alpha-induced protein 3 (*TNFAIP3*), CD69 molecule (*CD69*), SBDS ribosome assembly guanine nucleotide exchange factor (*SBDS*), and DNA-damage-inducible transcript 3 (*DDIT3*). Other significantly downregulated immune-related DEGs included *TNIP1*, *TNIP2, TNF, IFNG, IL10, IL12A*, as well as several genes related to MHC class 2 (*HLA*-*DRA, HLA*-*DQA2, HLA*-*DPB1, HLA*-*DMA*), and the nuclear factor kappa-light-chain-enhancer of activated B cells (NF-kappa-beta complex: *REL*, *RELA*, and *RELB*) (Table 1). Upregulated immune-related genes included *IL16* and *IL24* (encoding cytokines), *TLR1, TLR4, TLR5, TLR6, TLR7, TLR8*, and *TLR10* (encoding Toll-like receptors), and genes coding signal transducer and activator of transcription (STAT) proteins (Table 1). Also, some important apoptosis-related genes were upregulated, such as *BCL2*, *FASLG*, *MAF*, and *CASP2* as well as *CASP3*, *CASP6, CASP8*, and *CASP10*. We note that DNA (cytosine-5-)-methyltransferase 1 (*DNMT1*) was upregulated (log_2_FC = 0.59,* q* = 0.043), but none of the *de novo* DNA methyltransferases (*DNMT3A*, *DNMT3B*, and *DNMT3L*) was differently expressed.

**Table 1:** Selection of immune-related differentially expressed genes (DEGs) between high-arsenic (*n* = 4) and lower-arsenic (*n* = 4) exposure groups

Gene name	Gene description	Log_2_FC^a^	*Q* ^a^
*RELB*	v-rel avian reticuloendotheliosis viral oncogene homolog B	−1.83	4.1 × 10^−19^
*TNF*	Tumor necrosis factor	−2.70	5.2 × 10^−16^
*CASP3*	Caspase 3, apoptosis-related cysteine peptidase	1.70	2.8 × 10^−12^
*IFNG*	Interferon, gamma	−2.45	5.8 × 10^−10^
*TLR8*	Toll-like receptor 8	−2.65	1.0 × 10^−08^
*CASP10*	Caspase 10, apoptosis-related cysteine peptidase	0.98	2.6 × 10^−08^
*HLA*-*DQA2*	Major histocompatibility complex, class II, DQ alpha 2	−1.56	8.4 × 10^−08^
*TLR4*	Toll-like receptor 4	2.35	1.8 × 10^−06^
*TLR10*	Toll-like receptor 10	2.14	5.3 × 10^−06^
*BCL2*	B cell CLL/lymphoma 2	1.20	7.6 × 10^−06^
*CASP6*	Caspase 6, apoptosis-related cysteine peptidase	1.15	1.2 × 10^−05^
*TLR7*	Toll-like receptor 7	1.96	1.4 × 10^−05^
*MAF*	v-maf avian musculoaponeurotic fibrosarcoma oncogene homolog	1.16	4.4 × 10^−05^
*RELA*	v-rel avian reticuloendotheliosis viral oncogene homolog A	−0.98	0.0001
*IL12A*	Interleukin 12A	−2.04	0.0001
*STAT2*	Signal transducer and activator of transcription 2	0.98	0.001
*TLR5*	Toll-like receptor 5	1.63	0.002
*STAT5A*	Signal transducer and activator of transcription 5A	0.61	0.002
*REL*	v-rel avian reticuloendotheliosis viral oncogene homolog	−0.96	0.002
*IL24*	Interleukin 24	1.20	0.002
*IL16*	Interleukin 16	0.94	0.002
*TNIP1*	TNFAIP3 interacting protein 1	−0.59	0.003
*CASP8*	Caspase 8, apoptosis-related cysteine peptidase	1.00	0.003
*IL10*	Interleukin 10	−2.10	0.003
*STAT1*	Signal transducer and activator of transcription 1	1.15	0.003
*STAT3*	Signal transducer and activator of transcription 33response factor)	0.52	0.004
*TLR1*	Toll-like receptor 1	1.09	0.008
*FASLG*	Fas ligand (TNF superfamily, member 6)	1.83	0.008
*TNIP2*	TNFAIP3 interacting protein 2	−0.81	0.008
*HLA*-*DMA*	Major histocompatibility complex, class II, DM alpha	−0.83	0.015
*HLA*-*DPB1*	Major histocompatibility complex, class II, DP beta 1	−0.84	0.016
*TLR6*	Toll-like receptor 6	1.19	0.017
*CASP2*	Caspase 2, apoptosis-related cysteine peptidase	0.57	0.017
*HLA*-*DRA*	Major histocompatibility complex, class II, DR alpha	−0.85	0.029

The DEGs are sorted based on *p* value (immune genes presented in Table S2 are not included)

^a^ Log_2_FC = log-twofold change, *q* = FDR-adjusted *p* value

We then performed a gene ontology analysis with the topGO algorithm, considering the upregulated and downregulated DEGs separately and together. The top gene ontologies for the downregulated DEGs were related to RNA metabolism, including one category for viral transcription. The top gene ontology category for upregulated DEGs was regulation of transcription, DNA-templated. In general, categories related to RNA metabolism were significantly enriched among both the up- and downregulated DEGs, including general transcriptional and translational processes (Table S3).

### Methylomics of CD4-positive cells

For each exposure group, we used bisulfite sequencing to examine two technical replicates of pooled DNA from all individuals in each exposure group and evaluated the technical variability of the data by plotting the cumulative distribution of methylation (Figure S1). The two replicates were almost identical, indicating very little technical variability in the data. A clear shift between the plots of the high- and low-exposure groups suggested that a large number of CpG sites were differentially methylated between the exposure groups. To evaluate the epigenome-wide effect of arsenic exposure, we computed methylation levels for each site and compared the number of CpGs showing full methylation (defined as >80% methylation) between exposure groups. We found that in the two technical replicates of the lower-exposure group, 41.3 and 41.2% of the CpG sites showed full methylation, compared with 44.2% in both technical replicates of the high-exposure group, i.e., 3 percentage points higher full methylation, genome wide. DNA methylation increased for the high-exposure group at certain functional elements, particularly in CpG shelves, gene bodies, CpG shores, and gene promoters (Figure S2), but not in CpG islands.

**Fig. 2:**
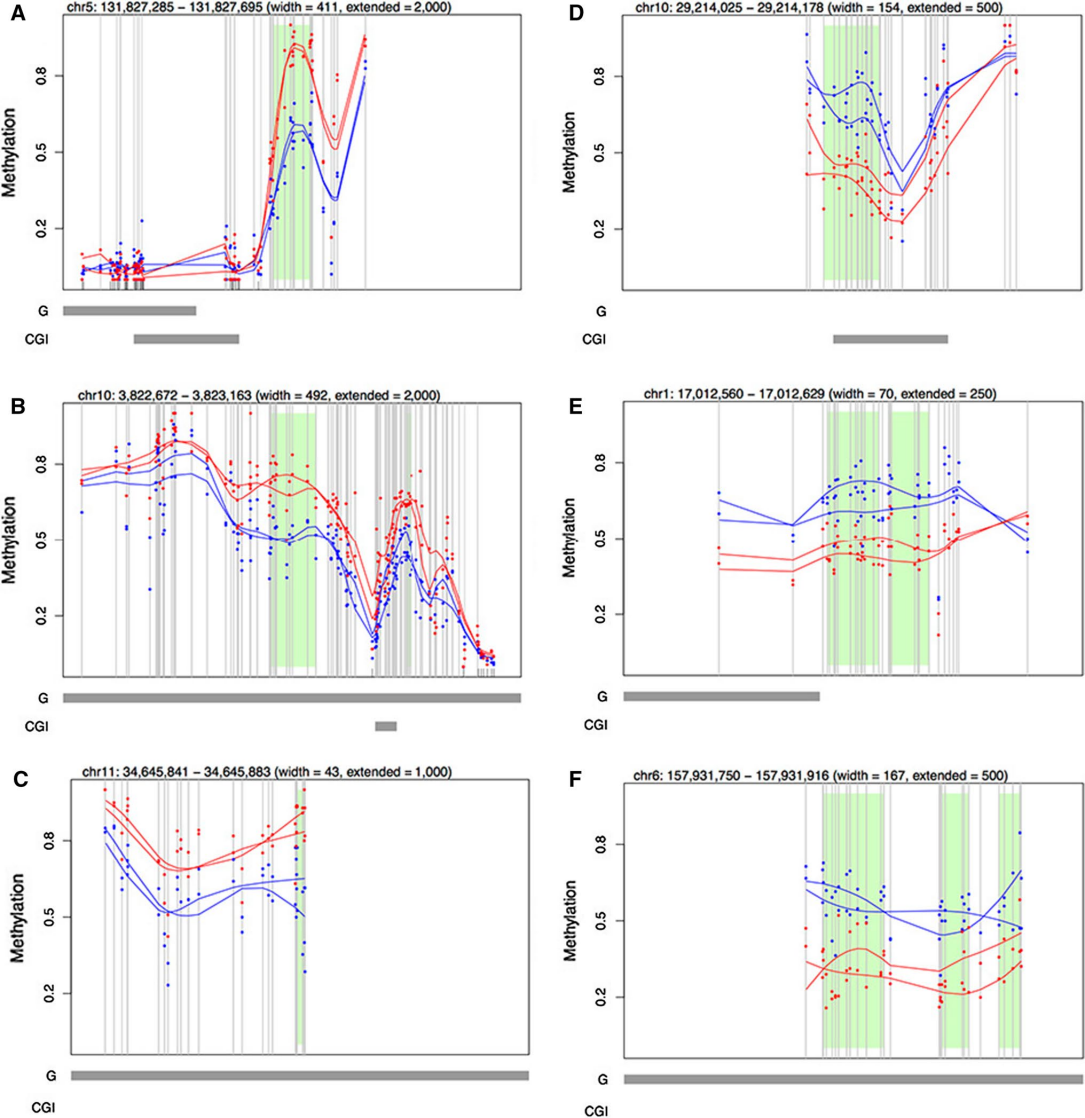
Single CpG resolution of differences in DNA methylation between arsenic exposure groups.** a**−**c** Differentially methylated regions (DMRs) hypermethylated in the high-arsenic exposure group; **e**−**g** DMRs hypomethylated in the high-arsenic exposure group. The chromosome number and genomic position are shown above the figure. Exposure groups are shown in *blue* (lower-arsenic exposure) and *red* (high exposure); *vertical bars* represent CpGs covered by targeted sequencing in specific regions. The methylation on the y-axis is shown as beta values. *Green boxes* are regions of the genome where DMRs (defined here as four consecutive CpG sites with a difference in methylation above 10% between high- and low-arsenic groups) were identified. *Bottom bars* indicate distances of the DMR to the closest gene (G) and CpG island (CGI)

We identified 1168 differentially methylated regions (DMRs) of which 391 (33.5%) showed hypermethylation and 777 (66.5%) hypomethylation in the high-exposure group. The top 10 DMRs are presented in Table S4, and examples of statistically significant methylation changes at a single-base resolution are shown in Fig. 2. Among the top 50 DMRs, two immune-related genes have previously been associated with arsenic exposure (Ramsey et al. 2013; Ding et al. 2016), encoding secretoglobin family 3A member 1 (*SCGB3A1*) and nuclear factor of activated T cells 1 (*NFATC1*). Both genes were hypomethylated in the high-arsenic group. The 1168 DMRs included several immune-related genes, such as interleukins *IL9, IL10, IL13*, *IL32* (hypermethylated)*, IL36A* (hypomethylated), members of the interferon regulatory factor (IRF) family; *IRF1, IRF4, IRF7,* and *IRF8* (all hypermethylated), members of the cluster of differentiation (CD) family *CD2, CD58* (hypermethylated)*, CD6, CD27, CD40* (hypomethylated), two genes related to MHC class (*HLA*-*DQAI*, which was hypermethylated, and *HLA*-*DRB5*, for which one DMR was hypermethylated and one DMR hypomethylated), as well as *IFNG* and *TLR10*.

The gene ontology analyses of hypermethylated DMRs (performed by the GREAT platform) showed an enrichment of cellular processes that relate to immune function of CD4-positive T cells, such as processes related to immunoglobulins and isotype switching (positive regulation of isotype switching, positive regulation of isotype switching to IgE isotypes, positive regulation of isotype switching to IgG isotypes, regulation of immunoglobulin production) and lymphocyte activation and differentiation (Table 2). By contrast, hypomethylated DMRs showed an enrichment of developmental processes, such as embryonic organ morphogenesis (Table 2). Hypermethylated DMRs also showed enrichment of genes related to V(D)J recombination.

**Table 2:** Top gene ontologies (GOs) related to differently methylated regions between high-arsenic (*n* = 4) and lower-arsenic (*n* = 4) exposure groups

GO ID	Term	*P* value
DMRs hypermethylated in high**-**arsenic exposure group		
GO:0045830	Positive regulation of isotype switching	5.25 × 10^−6^
GO:0045911	Positive regulation of DNA recombination	8.91 × 10^−6^
GO:0046649	Lymphocyte activation	1.45 × 10^−5^
GO:0048295	Positive regulation of isotype switching to IgE isotypes	2.95 × 10^−5^
GO:0002637	Regulation of immunoglobulin production	3.09 × 10^−5^
GO:0030098	Lymphocyte differentiation	3.89 × 10^−5^
GO:0006414	Translational elongation	4.07 × 10^−5^
GO:0002703	Regulation of leukocyte-mediated immunity	5.5 × 10^−5^
GO:0045087	Innate immune response	6.17 × 10^−5^
GO:0045191	Regulation of isotype switching	6.31 × 10^−5^
GO:0048304	Positive regulation of isotype switching to IgG isotypes	7.24 × 10^−5^
DMRs hypomethylated in high**-**arsenic exposure group		
GO:0048562	Embryonic organ morphogenesis	1.26 × 10^−9^
GO:0060741	Prostate gland stromal morphogenesis	1.78 × 10^−6^
GO:0035518	Histone H2A monoubiquitination	3.31 × 10^−6^
GO:0045668	Negative regulation of osteoblast differentiation	8.13 × 10^−6^
GO:0060684	Epithelial–mesenchymal cell signaling	9.55 × 10^−6^
GO:0033522	Histone H2A ubiquitination	1.29 × 10^−5^
GO:0070372	Regulation of ERK1 and ERK2 cascade	1.48 × 10^−5^
GO:0033151	V(D)J recombination	1.58 × 10^−5^
GO:0048701	Embryonic cranial skeleton morphogenesis	2.19 × 10^−5^
GO:0043433	Negative regulation of sequence-specific DNA binding transcription factor activity	2.19 × 10^−5^
GO:0010464	Regulation of mesenchymal cell proliferation	2.29 × 10^−5^
GO:0070374	Positive regulation of ERK1 and ERK2 cascade	2.82 × 10^−5^
GO:0010830	Regulation of myotube differentiation	3.16 × 10^−5^

To validate the accuracy of the methylome analysis, we analyzed four statistically significant DMRs using MS-HRM assays, and all confirmed a methylation difference between samples from high- and lower-exposure groups (data not shown).

### Alignment of DEGs and promoter DMRs associated with arsenic exposure group

In total, 32 genes had DMRs and DEGs that both statistically significantly associated with arsenic exposure group (top 10 genes in Table S5). Two of the 32 genes are involved in immune responses (Miyamoto et al. 1988; Dorn et al. 2007) (Figure S3): (1) interferon regulatory factor 1 (*IRF1*), for which the gene expression was downregulated and the DMR hypermethylated, and (2) ras homolog family member H (*RHOH*) for which the gene expression was downregulated and the DMR hypomethylated.

## Discussion

Our study suggests that chronic arsenic exposure through drinking water is related to changes in the transcriptome and methylome, both genome wide and for specific genes, of CD4-positive T cells in Andean women. Several key genes in the immune system were differently expressed at higher-arsenic exposure, and DMRs that were hypermethylated in the high-arsenic exposure group showed enrichment of immune-related processes that constitute the basic functions of CD4-positive T cells. These findings support the hypothesis that elevated arsenic exposure may cause immunotoxicity by interfering with gene expression and gene regulation. Still, we could not link many DMRs in promoter regions to changes in gene expression, which may be due to limited sequencing coverage, few samples analyzed and only partial overlap, or the arsenic-related changes in gene expression may be partly mediated by mechanisms other than DNA methylation.

### Arsenic exposure was associated with changes in genome-wide gene expression and expression of specific immune-related genes

Arsenic exposure was associated with genome-wide gene expression. In spite of the few samples analyzed, the first principal component, which separated samples based on their exposure group, explained more than half of the observed variance in gene expression for the top 20% variable genes and more than 5000 genes were differently expressed between the arsenic exposure groups. Among the top ten DEGs, four (all downregulated) have been linked to immune system function, and for three of these (*TNFAIP3*, *CD69*, and *DDIT3*), changes in expression have previously been associated with different types of arsenic exposure (Wang et al. 2006; Andrew et al. 2008; Li et al. 2013; Nadeau et al. 2014). The deubiquitinating enzyme TNFAIP3 (also called A20) is an important negative regulator of innate and adaptive immunity. TNFAIP3 participates in immune and inflammatory responses signaled by cytokines, such as TNF, and TNFAIP3 is rapidly induced by TNF (Dixit et al. 1990). TNFAIP3 also responds to signaling by Toll-like receptors (Boone et al. 2004) and affects immune responses by strongly inhibiting NF-kappa-B activity (Heyninck and Beyaert 2005). *TNFAIP3* is most highly expressed in T cells and, in particular, in CD4 positive T cells (http://genevisible.com/tissues/HS/UniProt/P21580). The downregulation of *TNFAIP3* is consistent with the downregulation of *TNF* with increasing arsenic exposure identified in this study, as well as with the downregulation of TNFAIP3-interacting proteins 1 and 2 (*TNIP1* and *TNIP2*). Also, *TNFAIP3* was upregulated in a multiple myeloma cell line after treatment with realgar, an arsenic sulfide mineral (Wang et al. 2006).

*CD69* codes for a surface marker expressed on CD4 positive T cells. The activation of T lymphocytes induces expression of *CD69*. CD69 appears to be the earliest inducible cell surface glycoprotein acquired during lymphoid activation and is involved in lymphocyte proliferation. CD69 functions as a signal-transmitting receptor in lymphocytes, including NK cells, and platelets (Cambiaggi et al. 1992). Downregulation of *CD69*, as observed here, could thus be linked to decreased activation of T lymphocytes. The downregulation of *CD69* is in line with the results from a population exposed to low levels of arsenic in New Hampshire (*N* = 21), where increasing arsenic exposure from drinking water was associated with decreased *CD69* expression in peripheral blood lymphocytes (Andrew et al. 2008). Another study in New Hampshire showed that in utero environmental arsenic exposure was inversely related to absolute total cord blood CD69 + T cell counts (Nadeau et al. 2014).

DNA-damage-inducible transcript 3 (*DDIT3*), also known as C/EBP-homologous protein (*CHOP*), encodes a transcription factor. *DDIT3* has previously been shown to be upregulated in CD4-positive T cells in mice in response to arsenic trioxide treatment (Li et al. 2013). The upregulation of *TNFAIP3* and *DDIT3* found in previous studies is in contrast to the downregulation of these genes observed in our study. However, the expression of these genes could be dose dependent, since the doses of arsenic trioxide or realgar were much higher than the doses from drinking water in the current study.

Furthermore, we found that several key players in the immune system were downregulated, such as *TNF*, *IFNG*, and *IL10*, as well as several genes related to MHC class 2 and the NF-kappa-B complex, while several key genes related to apoptosis were upregulated. Most of these findings are in accordance with previous studies investigating the association between gene expression (measured by microarray) and arsenic exposure in peripheral blood (Dangleben et al. 2013). Apoptosis is important for the homeostasis of the immune system, and abnormal immune cell apoptosis can contribute to dysregulation of immune function (Boise et al. 1995). The decrease in T cells with increasing arsenic exposure could be mediated by the upregulation of apoptotic genes. The regulation of mRNA levels of these genes may hypothetically be due to an effect of arsenic exposure on RNA metabolism, since several gene ontologies related to RNA metabolism were strongly enriched in the high-arsenic group.

### Exposure to arsenic influenced genome-wide and gene-specific 5-methylcytosine methylation

Our analysis of the methylome indicated that 3% of the genome-wide CpGs gained full methylation in the high-arsenic samples. By contrast, earlier in vitro and in vivo experiments associated arsenic exposure with global hypomethylation, measured as methylation of repetitive elements (Intarasunanont et al. 2012; Bailey and Fry 2014; Kile et al. 2014). Here, we measured methylation at CpG sites in regulatory sequences, which showed that these regions were hypermethylated in women in the high-arsenic exposure group. This suggests that in response to arsenic exposure, transcriptionally active parts of the genome gain methylation while repetitive elements lose methylation. Arsenic exposure did not appear to affect the methylation of CpG islands, but previous work showed that those elements are generally protected from changes in methylation (Deaton and Bird 2011). The increase in genome-wide DNA methylation may partly be explained by the upregulation of *DNMT1* expression in the high-arsenic exposure group, as DNMT1 is the major enzyme responsible for maintaining methylation patterns following DNA replication in somatic cells.

In addition, arsenic exposure also seemed to be associated with locus-specific DNA methylation. Two of the top 50 DMRs in the arsenic exposure group (both hypomethylated) covered immune-related genes that have previously been associated with gene expression in relation to arsenic. The secretoglobin gene *SCGB3A1* was upregulated in the lung in C57BL/6 mice exposed to arsenic in drinking water (Ramsey et al. 2013). Secretoglobins have anti-inflammatory and immunomodulatory functions (Jackson et al. 2011). *NFATC1* plays a central role in inducible transcription of cytokine genes in T cells (Chuvpilo et al. 1999) and was downregulated by arsenic in HCT116, a colon cancer cell line (Ding et al. 2016).

Gene ontology analysis of the DMRs that were hypermethylated in the high-arsenic group showed enrichment of immune-related processes that constitute the basic functions of CD4-positive T cells, such as isotype switching, especially the switch to IgE or IgG. Hypomethylated DMRs, by contrast, were linked to embryonic organ development, in particular. This is of interest, as a previous study found genome-wide hypomethylation in blood from newborns with elevated prenatal exposure to arsenic (Broberg et al. 2014).

### Differentially methylated regions in gene promoters correlated partly with gene expression

Thirty-two genes were significantly differentially expressed in the transcriptome analyses and also harbored a DMR. Two of the top genes, *IRF1* and *RHOH*, are involved in immune responses, and both were downregulated in the high-arsenic group. IRF1 is a member of the interferon regulatory transcription factor (IRF) family and activates transcription of interferons alpha and beta, as well as of genes induced by interferons alpha, beta, and gamma (Miyamoto et al. 1988). Thus, the downregulation of *IRF1* may be linked to the downregulation of *IFNG* seen in this study. IRF1 plays an important role in Th1 development and maturation of T helper cells (Lohoff and Mak 2005) and is involved in the suppression of regulatory T cell development (Fragale et al. 2008). RHOH is a critical regulator of thymocyte development and T cell antigen receptor signaling (Dorn et al. 2007).

### Strengths and limitations of the current study

The main strength of this study was that we used large-scale NGS on sorted CD4-positive T cells, which allowed us to conduct a genome-wide investigation of how this specific immune cell may respond to arsenic exposure. The technical variability in our sequencing data was minor, and the contrast in arsenic exposure was large; this enabled us to reveal group differences in the methylome and the transcriptome, despite the limited number of samples. However, further studies are needed for evaluation of causality as well as the dose–effect relationships. To note, the group with lower exposure was also somewhat exposed to arsenic; thus, there is no unexposed group in this study. A limitation of the study is the small number of samples and that the samples for the two different sequencing analyses were not completely overlapping, due to sample limitations. Also, only women were included; thus, we do not know whether these results also extend to men. Moreover, this is a unique population for studies on the effects of arsenic exposure, as they have an efficient metabolism of arsenic (Vahter et al. 1995), known to be related to lower risk of toxicity This population has been exposed to arsenic in drinking water for thousands of years and likely has adapted to tolerate arsenic (Schlebusch et al. 2013, 2015). Thus, the indicated changes in gene expression and DNA methylation may not necessarily be considered toxic responses, since they may occur following an adaptive response, e.g., acquired resistance over time. Thus, the effects in a population without this background may differ from the effects found in this study. Further and larger studies, in other populations with wide ranges in chronic arsenic exposure, and also including men, will be needed to validate the results.

## Conclusions

We showed that chronic arsenic exposure from drinking water is related to changes in the transcriptome and methylome of CD4-positive T cells, both genome wide and for specific genes, supporting the hypothesis that arsenic causes immunotoxicity by interfering with gene expression and gene regulation.

## Electronic supplementary material

Below is the link to the electronic supplementary material.

Supplementary material 1 (PDF 2338 kb)
