# Supplementary material for: Transcriptomics and methylomics of CD4-positive T cells in arsenic-exposed women
Source: Arch Toxicol. 2016 Nov 12;91(5):2067–78. doi: 10.1007/s00204-016-1879-4 (PMC5399044; doi:10.1007/s00204-016-1879-4)
Supplement: Supplementary file 1 — Supplementary material 1 (PDF 2338 kb) [file 204_2016_1879_MOESM1_ESM.pdf]

Supplemental material for:

Transcriptomics and Methylomics of CD4-Positive T Cells in Arsenic-Exposed Women

Karin Engström[1,2*], Tomasz K Wojdacz [2*] Francesco Marabita [3, 4], Philip Ewels [5], Max Käller [6], Francesco Vezzi [5], Nicola Prezza [6], Joel Gruselius [7], Marie Vahter [1], Karin Broberg[2]

[1]Department of Laboratory Medicine, Division of Occupational and Environmental Medicine, Lund University, 221 85 Lund, Sweden

[2]Unit of Metals and Health, Institute of Environmental Medicine, Karolinska Institutet, 171 77 Stockholm, Sweden

[3]Unit of Computational Medicine, Center for Molecular Medicine, Karolinska University Hospital L805, 171 76 Stockholm, Sweden

[4]Bioinformatics Infrastructure for Life Sciences, Karolinska Institutet, 171 65 Solna, Sweden

[5]Science for Life Laboratory (SciLifeLab), Department of Biochemistry and Biophysics, Stockholm University, 106 91 Stockholm, Sweden

[6]Science for Life Laboratory, School of Biotechnology, Division of Gene Technology, Royal Institute of Technology, 171 65 Solna, Sweden

[7]Department of Mathematical and Computer Science, University of Udine, 331 00 Udine, Italy

* These authors contributed equally to the work and are listed in alphabetic order.

Table of contents

Table S1. Characteristics of the women in the study.

Table S2. Top ten differentially expressed genes (DEGs) (based on p-value) between arsenic exposure groups.

Table S3. List of the top 10 gene ontologies related to arsenic exposure.

Table S4. The top 10 differentially methylated regions (DMRs) comparing lower (reference) and high arsenic exposure groups.

Table S5. Top 10 genes with both differently methylated and differently expressed genes related to arsenic exposure.

Figure S1. Cumulative distribution plots of each of the technical replicates tested for methylomics analysis.

Figure S2. Differences in DNA methylation of specific genomic regions between arsenic exposure groups.

Figure S3. Single CpG resolution of differences in DNA methylation between arsenic exposure groups from two genes that were significantly associated with both DNA methylation and gene expression.

Table S1. Characteristics of the women in the study[a]

Variable	Exposure group	Transcriptomics (median, range)	Methylomics (median, range)	P-value[b]
Age	All	32 (27-52)	39 (22-56)	0.53
	Lower exposed	44 (27-52)	51 (22-56)	
	High exposed	30.5 (28-33)	35 (24-42)	
p-value[c]		0.34	0.34	
BMI (kg/m^2)	All	27 (24-35)	27 (21-30)	0.53
	Lower exposed	28 (24-35)	27 (22-30)	
	High exposed	26 (25-30)	26 (21-29)	
p-value[c]		0.69	0.48	
Coca (percent users)	All	62.5%	100%	0.20
	Lower exposed	50%	100%	
	High exposed	75%	100%	
p-value[c]		1.0	1.0	
Total urinary arsenic (µg/L)[d]	All	135 (48-604)	160 (48-604)	0.96
	Lower exposed	65 (48-77)	59 (48-69)	
	High exposed	276 (193-604)	301 (252-604)	
p-value[c]		0.029	0.029	

[a] N=8 for the "All" groups, N=4 for each of the low and high exposure groups, respectively. The overlap between the groups analysed for transcriptomics and methylation is three individuals.

[b] P-value for the difference between the individuals included in transcriptomics and methylomics analyses (Mann-Whitney test was employed for age, BMI and total urinary arsenic, while Fishers exact test was employed for coca)

[c] P-value for the difference between the high and low exposure group (Mann-Whitney test was employed for for age, BMI and total urinary arsenic, while Fishers exact test was employed for coca)

[d] Calculated as the sum of arsenic metabolites (inorganic arsenic, methylarsonic acid, and dimethylarsinic acid) adjusted to the average specific gravity of 1.020 g/mL .

Table S2. Top ten differentially expressed genes (DEGs) (based on p-value) between arsenic exposure groups.

Gene	Gene description	Log_2FC	q
TNFAIP3	tumor necrosis factor, alpha-induced protein 3	-3.73	$1.6*10^{-101}$
CD69	CD69 molecule	-3.59	$2.1*10^{-77}$
TP53INP2	tumor protein p53 inducible nuclear protein 2	-3.91	$7.2*10^{-62}$
SBDS	SBDS ribosome assembly guanine nucleotide exchange factor	-2.79	$9.5*10^{-52}$
NINJ1	ninjurin 1	-2.72	$2*10^{-51}$
WHAMM	WAS protein homolog associated with actin, golgi membranes and microtubules	-2.82	$4.3*10^{-50}$
DDIT3	DNA-damage-inducible transcript 3	-3.03	$1.5*10^{-43}$
GRASP	GRP1 (general receptor for phosphoinositides 1)-associated scaffold protein	-3.56	$5.1*10^{-42}$
MIDN	midnolin	-2.30	$1.8*10^{-41}$
DUSP8	dual specificity phosphatase 8	-4.44	$2.1*10^{-41}$

q= FDR-adjusted p-value, Log_2FC = log-2 fold change.

Table S3. List of the top ten gene ontologies related to arsenic exposure.

GO ID	Term	No. of annotated genes in GO	No. of DEGs in GO	No. of expected DEGs in GO	P value
All DEGs					
GO:0006414	translational elongation	194	114	66	$1.7*10^{-20}$
GO:0019083	viral transcription	179	110	61	$4.0*10^{-19}$
GO:0006614	SRP-dependent cotranslational protein targeting to membrane	105	79	36	$5.0*10^{-18}$
GO:0006415	translational termination	170	100	58	$5.3*10^{-18}$
GO:0000184	nuclear-transcribed mRNA catabolic process, nonsense-mediated decay	114	82	39	$1.1*10^{-16}$
GO:0006355	regulation of transcription, DNA-templated	2535	1032	862	$1.7*10^{-15}$
GO:0006413	translational initiation	250	138	85	$1.1*10^{-14}$
GO:0006357	regulation of transcription from RNA polymerase II promoter	1191	460	405	$6.2*10^{-05}$
GO:0051276	chromosome organization	823	301	280	0.0003
GO:0006364	rRNA processing	136	76	46	0.0003
Upregulated DEGs (in high arsenic exposure group compared to low arsenic exposure group)					
GO:0006355	regulation of transcription, DNA-templated	2535	546	485	$5.1*10^{-17}$
GO:0010827	regulation of glucose transport	74	22	14	$8.3*10^{-05}$
GO:0008033	tRNA processing	96	38	18	$8.5*10^{-05}$
GO:0030488	tRNA methylation	15	9	3	0.0006
GO:0006606	protein import into nucleus	195	40	37	0.0007
GO:0015031	protein transport	1408	280	269	0.0008
GO:0006418	tRNA aminoacylation for protein translation	46	20	9	0.0008
GO:0007077	mitotic nuclear envelope disassembly	42	17	8	0.001
GO:0006189	'de novo' IMP biosynthetic process	6	5	1	0.001

GO:0051028	mRNA transport	118	34	23	0.001
Downregulated DEGs (in high arsenic exposure group compared to low arsenic exposure group)					
GO:0006414	translational elongation	194	87	29	$< 1*10^{-30}$
GO:0006415	translational termination	170	78	25	$< 1*10^{-30}$
GO:0006614	SRP-dependent cotranslational protein targeting to membrane	105	74	16	$< 1*10^{-30}$
GO:0006413	translational initiation	250	103	37	$< 1*10^{-30}$
GO:0000184	nuclear-transcribed mRNA catabolic process, nonsense-mediated decay	114	74	17	$< 1*10^{-30}$
GO:0019083	viral transcription	179	84	27	$1.8*10^{-29}$
GO:0000122	negative regulation of transcription from RNA polymerase II promoter	503	122	75	$2.2*10^{-07}$
GO:0045893	positive regulation of transcription, DNA-templated	887	205	132	$6.9*10^{-06}$
GO:0034097	response to cytokine	523	105	78	$2.7*10^{-05}$
GO:1900102	negative regulation of endoplasmic reticulum unfolded protein response	9	7	1	$4.3*10^{-05}$

Table S4. The top 10 differentially methylated regions (DMRs) comparing lower (reference) and high arsenic exposure groups.

Gene	Gene description	Direction[a]	Area of DMR	Nr of CpGs in DMR
MIR5087	microRNA 5087	-	3.27	19
C19orf35	chromosome 19 open reading frame 35	-	3.03	19
FAM168B	family with sequence similarity 168, member B	-	2.91	15
SCGB3A1	secretoglobin, family 3A, member 1	-	2.72	16
CLEC3B	C-type lectin domain family 3, member B	-	2.66	15
LINC00837	long intergenic non-protein coding RNA 837	-	2.36	15
TMEM72	transmembrane protein 72	-	2.34	12
LOC728323	hypothetical LOC728323	+	2.30	14
PVT1	Pvt1 oncogene (non-protein coding)	-	2.19	14
MIR106A	microRNA 106a	+	2.17	14

[a] Direction of association with arsenic group (low vs higher arsenic), using low exposure group as a reference.

Table S5. Top 10 genes with both differently methylated and differently expressed genes related to arsenic exposure.

Gene	Gene description	Chromosome	DMR		DEG	
			Direction[a]	Area of DMR	Log_2FC	q
ZNF200	zinc finger protein 200	16	+	0.59	2.46	$3.5*10^{-18}$
PVT1	Pvt1 oncogene (non-protein coding)	8	-	2.19	-1.24	$2.9*10^{-08}$
SNX1	sorting nexin 1	15	-	0.70	1.38	$3.6*10^{-08}$
CYP2U1	cytochrome P450, family 2, subfamily U, polypeptide 1	4	-	0.51	1.54	$2.2*10^{-07}$
IRF1	interferon regulatory factor 1	5	+	2.02	-1.83	$2.5*10^{-07}$
C1GALT1C1	C1GALT1-specific chaperone 1	X	-	0.83	1.40	$3.4*10^{-07}$
ZNF696	zinc finger protein 696	8	-	0.65	2.84	$3.6*10^{-07}$
FAM43A	family with sequence similarity 43, member A	3	+	0.46	1.92	$1.3*10^{-06}$
FAM50B	family with sequence similarity 50, member B	6	-	1.21	2.03	$1.9*10^{-06}$
RHOH	ras homolog family member H	4	-	0.68	-1.40	0.00002

DEG = differently expressed genes, DMR = differentially methylated regions, q= FDR-adjusted p-value, Log_2FC = log-2 fold change

[a] Denotes the direction of the DMR analyses in relation to increasing arsenic exposure.

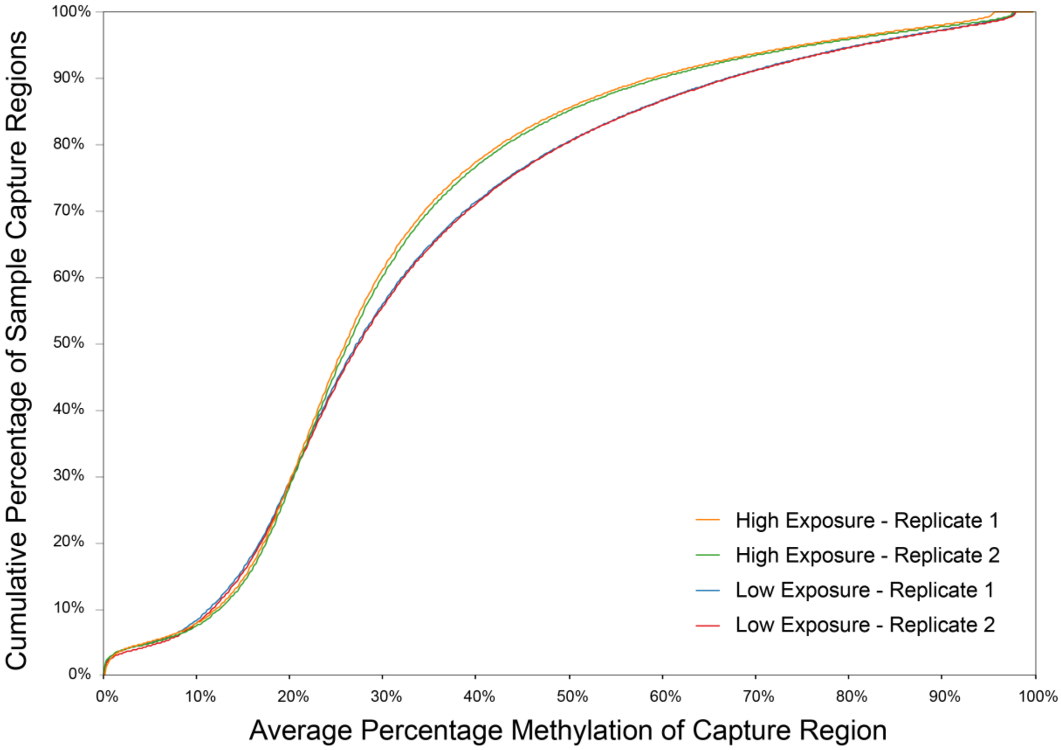

Figure S1. Cumulative distribution plots of each of the technical replicates tested for the methylomics analysis.

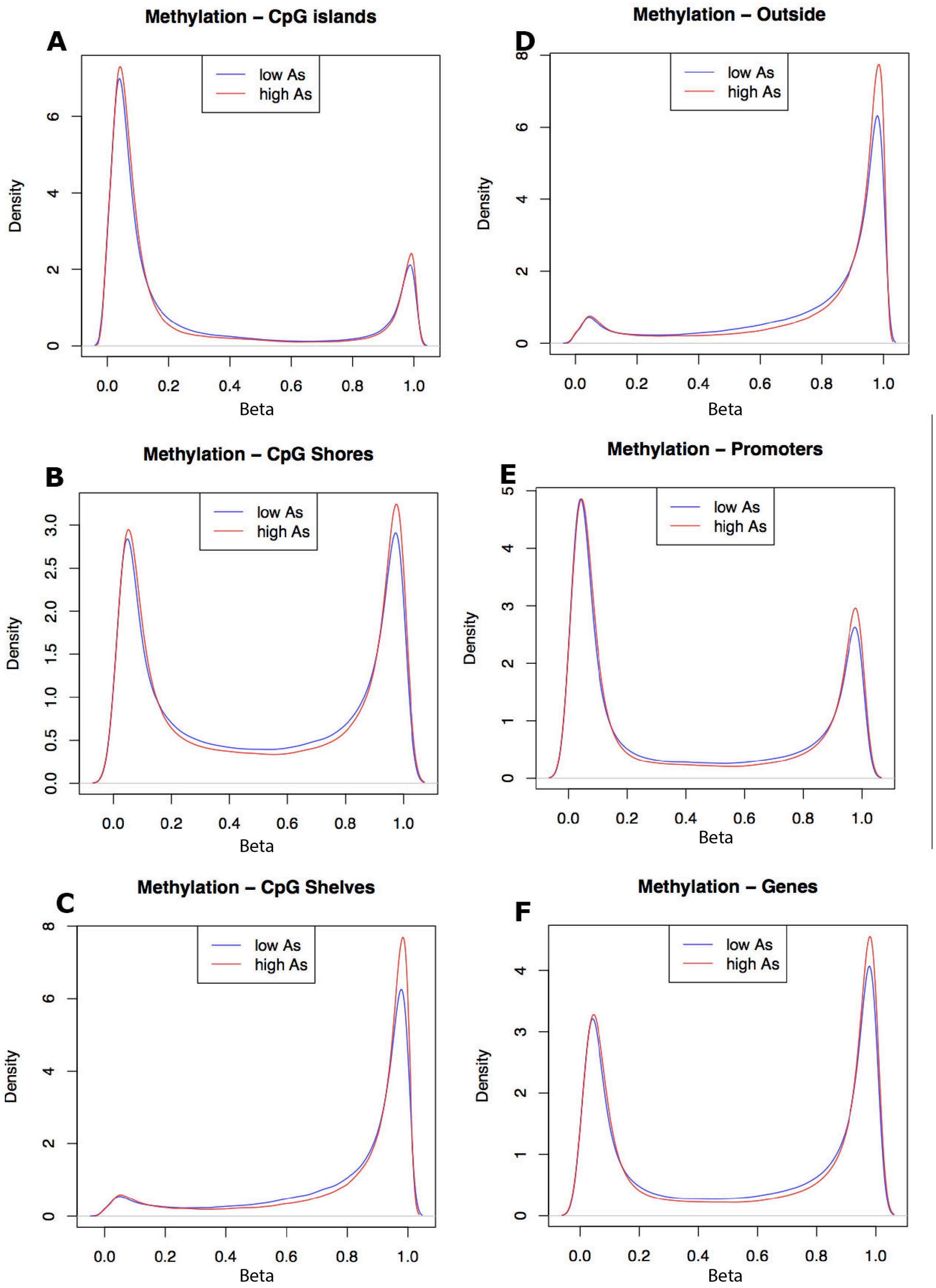

Figure S2. Differences in DNA methylation of specific genomic regions between arsenic exposure groups. Density plots describe mean methylation levels (beta-value) on the x-axis for specific genomic regions (indicted at the top of the figure) for lower (blue) and high (red) exposure groups ("outside" – refers to CpGs outside of CpG islands).

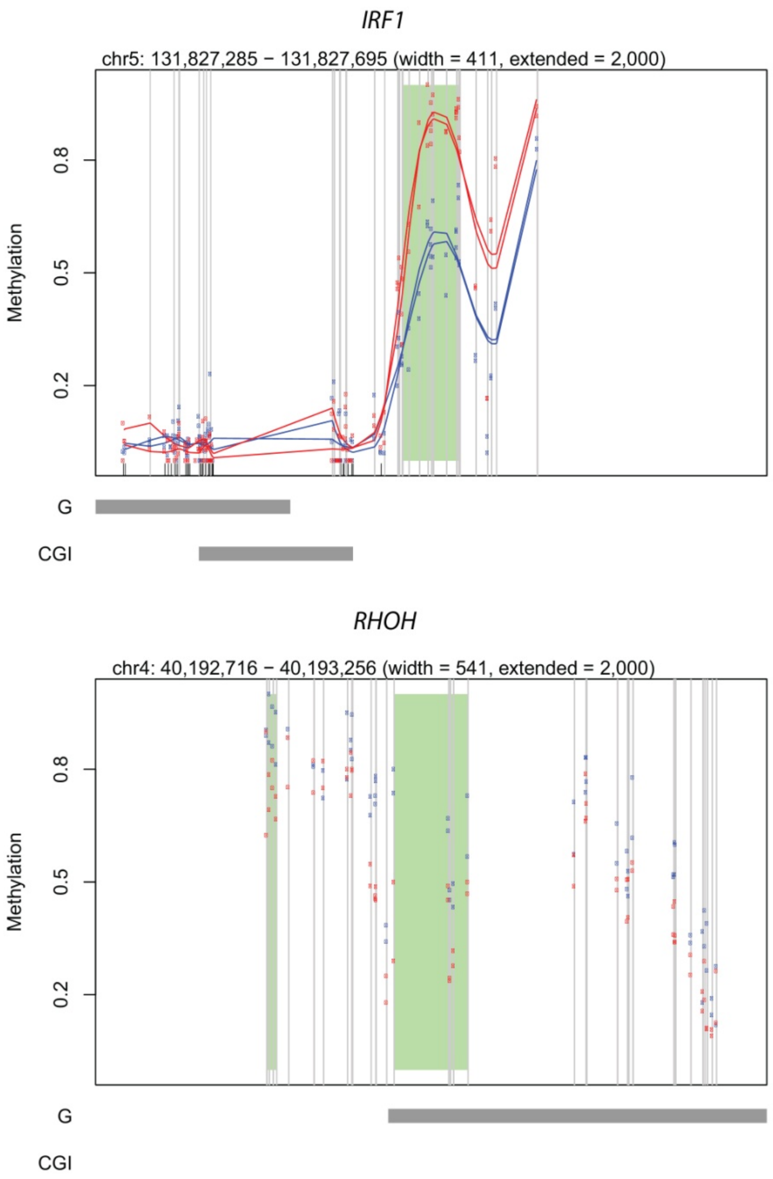

Figure S3. Single CpG resolution of differences in DNA methylation between arsenic exposure groups from two genes that were significantly associated with both DNA methylation and gene expression. The chromosome number and genomic position is shown above the figure. Exposure groups are shown in blue (lower exposure) and red (high exposure), vertical bars represent CpGs covered by targeted sequencing in specific regions. The methylation on the y-axis is shown as beta-values. Green boxes are regions of the genome where DMRs (defined here as four consecutive CpG sites with a difference in

methylation above 10% between high- and low-arsenic groups) were identified. Bottom bars indicate distances of the DMR to the closest gene (G) and CpG island (CGI).
